# The volatile oil of *Acorus tatarinowii* schott significantly attenuates neuroinflammatory damage in a rat model of tourette syndrome by inhibiting the p38 MAPK/NLRP3/STAT3 signaling pathway

**DOI:** 10.3389/fphar.2025.1540092

**Published:** 2025-09-25

**Authors:** Xing Wei, Kexin Sun, Peng Feng, Shuang Huang, Bing Jiang, Min Cui, Yuanhuan Chen, Fuchun Xue, Yuezhen He, Jingxi Yao, Jing Shang, Hao Mei, Yanqing Ding, Yongyan Tian, Guangyu Guo, Nan Yang, Zhenggang Shi

**Affiliations:** ^1^ Medical College, Hexi University, Zhangye, Gansu, China; ^2^ School of Clinical Chinese Medicine, Gansu University of Chinese Medicine, Lanzhou, Gansu, China; ^3^ Department of Integrated Chinese and Western medicine, Gansu University of Chinese Medicine, Lanzhou, Gansu, China; ^4^ Department of Traditional Chinese Medicine, The First Hospital of Lanzhou University, Lanzhou, Gansu, China; ^5^ Department of Pediatrics, Zhangye People’s Hospital Affiliated to Hexi University, Zhangye, Gansu, China; ^6^ Research Center for Silk Road Traditional Chinese Medicine, Hexi University, Zhangye, Gansu, China; ^7^ School of Basic Medical, Gansu University of Chinese Medicine, Lanzhou, Gansu, China

**Keywords:** volatile oil from *Acorus tatarinowii*, Tourette syndrome, neuroinflammation, p38 MAPK/NLRP3/STAT3, a rat model

## Abstract

**Background:**

*Acorus tatarinowii* Schott holds a prominent position in Traditional Chinese Medicine, with its earliest record found in the ancient Chinese pharmacopeia, the *Shennong’s Classic of Materia Medica*. It has been widely used for various central nervous system diseases. VOA has been shown to reduce neuroinflammation and repair neurons. However, the *in vivo* mechanisms by which this volatile oil alleviates neuroinflammation caused by Tourette syndrome remain unclear.

**Purpose:**

This study aims to investigate the effects and molecular mechanisms of VOA intervention in TS, providing scientific evidence for the potential therapeutic role of VOA in TS and paving the way for new treatment strategies.

**Methods:**

Forty-eight 3-week-old standard deviation rats were divided into a Blank group (n = 8) and a Model group (n = 40). After establishing the Tourette Syndrome animal model, the Model group rats were randomly divided into the Model, Tiapride, VOA, SB203580 and VOA + SB203580 groups. Following model induction, the respective treatments were administered continuously for 4 weeks. At the end of the intervention, Nissl staining was used to observe neuronal structure, and Enzyme-Linked Immunosorbent Assay, immunofluorescence, immunohistochemistry, RT-qPCR and WB were performed to determine the levels of inflammatory factors and protein expression.

**Results:**

Nissl staining showed that VOA significantly improved neuronal structure compared to the Model group. Compared to the Model group, the Tiapride, VOA, SB203580 and VOA + SB203580 groups had significantly reduced levels of TNF-α, IL-6, CD11b, COX-2, caspase-1, p38 MAPK, p-p38 MAPK, STAT3, p-STAT3, NLRP3, and GSDMD (*P* < 0.01 or *P* < 0.05) and significantly increased levels of IL-10 and CD163 (*P* < 0.01 or *P* < 0.05).

**Conclusion:**

VOA significantly alleviates neuroinflammation in TS rats by modulating the activity of the p38 MAPK/NLRP3/STAT3 signaling pathway, thereby improving the pathological characteristics of TS. These findings suggest that VOA could be a potential candidate for treating TS and other neuroinflammation-related diseases.

## Graphical Abstract

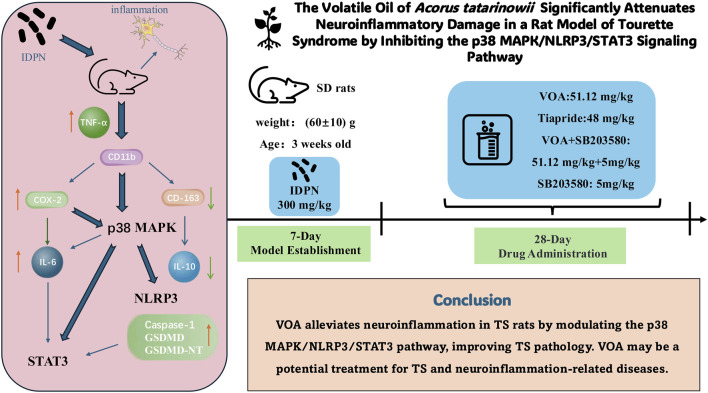

## Highlights

• VOA inhibits the p38 MAPK/NLRP3/STAT3 pathway, reducing neuroinflammation in TS rat.
• VOA repairs and protects neurons by regulating molecules like IL-6, IL-10, and COX-2.
• The experimental design employs multiple techniques assess VOA’s efficacy.
• VOA shows potential as a treatment for TS and other neuroinflammatory diseases.

## 1 Introduction

Tourette Syndrome (TS) is a chronic neuropsychiatric disorder commonly seen in childhood. It is primarily marked by involuntary, repetitive, quick, and purposeless muscle or vocal tics, which can be accompanied by emotional disturbances, hyperactivity, attention deficits, compulsive actions or thoughts, along with other behavioral issues (Hongyan et al., 2019). Various tic disorders (TD), including provisional tic disorder (PTD) and chronic motor or vocal tic disorder (CTD), fall under this classification, with TS being the most severe form (Müller-Vahl et al., 2019; Liu et al., 2020). Presently, TS treatment is categorized into behavioral and pharmacological therapies. Among behavioral approaches, only Comprehensive Behavioral Intervention for Tics (CBIT) is specifically endorsed by the American Academy of Neurology as effective. Nonetheless, its high cost restricts its widespread application. Pharmacological treatments mainly include atypical antipsychotics like aripiprazole, tiapride, and risperidone. Of note, tiapride is more commonly prescribed for children and adolescents compared to adults, often with favorable outcomes. However, prolonged use of these medications can result in extrapyramidal side effects such as muscle rigidity, tremors, and involuntary movements, along with drowsiness, nausea, increased prolactin levels, and weight gain. Although deep brain stimulation is employed for treatment-resistant cases, its limited sample size, varied study designs, and high costs hinder its broad implementation (Roessner et al., 2022; Andrén et al., 2022; Johnson et al., 2023). Therefore, there is a critical need to discover a safe and effective therapeutic approach for TS.

A growing body of evidence indicates a strong link between inflammatory cytokines and the development of TS, leading to significant alterations in serum and striatum levels of cytokines such as interleukin-6 (IL-6), interleukin-10 (IL-10), and tumor necrosis factor-alpha (TNF-α) (Long et al., 2019). Neuroinflammation, which is triggered by inflammatory stimuli, activates microglia within the central nervous system (CNS) as a fundamental protective mechanism. However, sustained neuroinflammation can result in neuronal dysfunction and even cell death Cherry (Cherry et al., 2014). Furthermore, neuroinflammation plays a critical role in a range of CNS disorders, including TS, autism spectrum disorder (ASD), Alzheimer’s disease (AD), and Parkinson’s disease (PD) (Mirarchi et al., 2023). Thus, mitigating the inflammatory response induced by overactive microglia and preventing subsequent cellular damage, alongside regulating neuroinflammatory processes, might be effective strategies for managing neuroinflammation and CNS disorders (Bachiller et al., 2018; Lim et al., 2018; Fatoba et al., 2020).

*Acorus tatarinowii* schott (The plant name “*A. tatarinowii*” has been verified through Kew Medicinal Plant Names Services (MPNS) and http://www.worldfloraonline.org. The plant name corresponds with the latest revision of “World Flora Online”. First published in Oesterr. Bot. Z. 9: 101 (1859). This name is a synonym of *Acorus verus*), commonly known as Shi Chang Pu, a traditional Chinese medicinal botanical drug first recorded in the “*Shennong Ben Cao Jing*,” possesses pharmacological properties such as sedation, anticonvulsant, antioxidant, anti-neuroinflammation, neurotransmitter regulation, and anti-apoptosis (Balakrishnan et al., 2022; Kim et al., 2022; Li et al., 2022; Tao et al., 2022; Wang M. et al., 2023). It has been widely used in treating neurological diseases (Zhang et al., 2019). Relevant pharmacological studies indicate that *A. tatarinowii* contains various metabolites, including volatile oils, flavonoids, alkaloids, and organic acids, with volatile oils being the primary active metabolites (Ma et al., 2015). Common extraction methods for volatile oils from *A. tatarinowii* include steam distillation, microwave-assisted extraction, ultrasonic extraction, and supercritical fluid extraction (Aziz et al., 2018; Bai et al., 2022). Several studies have identified its main active metabolites as α-asarone, β-asarone, methyleugenol, and caryophyllene (Limón et al., 2009; Wang et al., 2014; Lam et al., 2016; Shi et al., 2021). Numerous studies have confirmed that these metabolites, as important neuroprotective agents, can penetrate the blood-brain barrier, maintain the balance of neurotransmitters in the brain by adjusting excitatory and inhibitory responses, reduce neuroinflammatory responses, and effectively inhibit microglia-mediated neuroinflammatory responses by suppressing the production of pro-inflammatory cytokines (Chellian et al., 2017; Lee et al., 2018; Zhang et al., 2019; Ullah et al., 2021). Consequently, they exhibit neuroprotective effects (Balakrishnan et al., 2022; Hongyan et al., 2019; Kim et al., 2022; Ning et al., 2024), indicating positive potential for the prevention and treatment of CNS diseases (Shi et al., 2021; Bai et al., 2022). Therefore, these findings suggest that VOA may be an effective candidate for treating TS. Tiapride is one of the ten most commonly used antipsychotic drugs in pediatrics, particularly as a first-line treatment for children and adolescents with Tourette syndrome (Chellian et al., 2017; Roessner et al., 2022). It is widely used as a first-line medication for treating TS in Asian countries (Liu et al., 2020). Tiapride primarily exerts its therapeutic effects by blocking dopamine D2 receptors in the brain, with significant action in the striatum, which helps reduce extrapyramidal side effects. However, it can also cause adverse reactions such as dizziness, fatigue, drowsiness, and central nervous system effects, as well as gastrointestinal discomfort like nausea and vomiting (Roessner et al., 2022; Johnson et al., 2023). Therefore, we selected Tiapride as a positive control drug to evaluate the therapeutic potential of VOA in TS patients.

Although The Volatile Oil of *A. tatarinowii* (VOA) has shown potential therapeutic effects in the treatment of neuroinflammation and CNS diseases, its regulatory effects on neuroinflammatory damage in a TS rat model have not been fully explored. In particular, whether VOA can alleviate neuroinflammatory damage in TS model rats by the p38 MAPK/NLRP3/STAT3 signaling pathways requires systematic experimental investigation. In our previous studies (Feng et al., 2024), we first evaluated the therapeutic effects of different doses of VOA on a TS rat model, and the results demonstrated that High-dose VOA exhibited significant anti-tic effects. Based on these findings, the current study further focuses on the High-dose VOA group to investigate its potential in repairing neuroinflammatory damage by examining its inhibitory effects on the p38 MAPK/NLRP3/STAT3 signaling pathways.

## 2 Materials and methods

### 2.1 Materials

The Nissl staining kit was purchased from Beijing Solarbio Science & Technology Co., Ltd. (No. 1432, Beijing, China), xylene from Tianjin Bai Chen Fang Zheng Reagent Factory (Tianjin, China), hematoxylin from Beijing Solarbio Science & Technology Co., Ltd. (No. 1140, Beijing, China), neutral balsam from Beijing Solarbio Science & Technology Co., Ltd. (No. 8590, Beijing, China), hydrogen peroxide blocking solution from Beijing Solarbio Science & Technology Co., Ltd. (Beijing, China), IL-10 ELISA kit from Shanghai Enzyme-linked Biotechnology Co., Ltd. (No. MI037371, Shanghai, China), IL-6 ELISA kit from Shanghai Enzyme-linked Biotechnology Co., Ltd. (No. MI064292, Shanghai, China), TNF-α ELISA kit from Shanghai Enzyme-linked Biotechnology Co., Ltd. (No. MI002859, Shanghai, China), Caspase-1 antibody from Bioss Antibodies Inc. (No. bs-10442R, Beijing, China), CD163 antibody from Genetex (No. GTX42367, United States), COX-2 antibody from Santa Cruz Biotechnology (No. sc-166475, United States), CD11 b antibody from Bioss Antibodies Inc. (No. bs-1014R, Beijing, China), p38 MAPK antibody from Bioss Antibodies Inc. (No. bsm-33423M, Beijing, China), p-p38 MAPK antibody from Bioss Antibodies Inc. (No. bs-5476R, Beijing, China), STAT3 antibody from ImmunoWay Biotechnology Company (No. YM3641, United States), p-STAT3 antibody from ImmunoWay Biotechnology Company (No. YM3507, United States), NLRP3 antibody from Bioss Antibodies Inc. (No. bs-10021R, Beijing, China), rabbit SP kit from Beijing Zhong Shan Golden Bridge Biotechnology Co., Ltd. (No. SP-9001, Beijing, China), DAB staining kit from Beijing Zhong Shan Golden Bridge Biotechnology Co., Ltd. (No. K193328E, Beijing, China), TRIeasyTM Total RNA Extraction Reagent from Yuan Sheng Bio-Tech (Shanghai) Co., Ltd. (No. 10606ES60, Shanghai, China), HyperScriptTM RT SuperMix for RT-qPCR with Gdna Remover from Weihai Xinbeisi Biotechnology Co., Ltd. (No. R202-02, Shandong, China), 2×S6 Universal SYBR RT-qPCR Mix from Weihai Xinbeisi Biotechnology Co., Ltd. (No. Q204-01, Shandong, China), SDS-PAGE gel preparation kit from Beyotime Biotechnology Co., Ltd. (No. P0012A, Shanghai, China), BCA Protein Assay Kit from Beijing Solarbio Science & Technology Co., Ltd. (No. PC0020, Beijing, China), RIPA Lysis Buffer from Beijing Solarbio Science & Technology Co., Ltd. (No. R0010, Beijing, China), and goat anti-IgG from ImmunoWay Biotechnology Company (No. RS0002, United States).

### 2.2 Methods

#### 2.2.1 Preparation of drugs

Tiapride (produced by Jiangsu Enhua Pharmaceutical Co., Ltd., batch number: LY211202, specification: 100 mg/tablet) was ground and dissolved in saline to prepare a 3.194 mg/mL solution. *Acorus tatarinowii* (purchased from Sichuan Hongkangyuan Pharmaceutical Co., Ltd., batch number: 230401). The Volatile Oil of *A. tatarinowii* (VOA, Extraction was carried out using Method A as described in the General Rule 2204 for the Determination of Volatile Oils in the Chinese Pharmacopoeia, 2020 edition, Volume IV.) was dissolved in Tween-80 aqueous solution (1.25 mL Tween-80 in 100 mL ultrapure water) to prepare a 5 mg/mL solution. 3,3′-Iminodipropionitrile (IDPN, Shanghai Macklin Biochemical Co., Ltd., batch number: C14426981, specification: 25 g/bottle) was dissolved in saline to prepare a 30 mg/mL solution. SB203580 (Adezmapimod, MedChemexpress Biotech Co., United States, batch number: 159461, specification: 500 mg/bottle) was prepared as a 5 mg/kg solution and stored at −20°C.

#### 2.2.2 Preparation of volatile oils of *Acorus tatarinowii*

According to Method A in the “Determination of Volatile Oils” (General Rule 2204) of the Chinese Pharmacopoeia (2020 edition, Volume IV), the method for extracting volatile oil from *A. tatarinowii* is as follows: Pulverize the sample, take an appropriate amount (containing approximately 0.5–1.0 mL of volatile oil), and place it into a 1000 mL flask. Add 300–500 mL of water and glass beads to prevent bumping. Connect the volatile oil determination apparatus to a condenser, heat until boiling, and collect the volatile oil and water mixture until no more volatile oil is produced. After cooling to room temperature, the volume of the collected volatile oil was recorded, and its content was calculated as a percentage of the sample weight.

#### 2.2.3 Identification of volatile metabolites by GC-MS

The extracted volatile oil sample of *A. tatarinowii* was subsequently subjected to gas chromatography-mass spectrometry (GC-MS) analysis (Yuan, et al., 2022). Sample desorption was performed in the injection port of the gas chromatograph (Agilent, China) at 250°C for 5 min in splitless mode. GC-MS analysis was conducted using an Agilent model 8890 GC and a 7000D mass spectrometer (Agilent, China) equipped with a DB-5MS capillary column (30 m × 0.25 mm × 0.25 µm; 5% phenyl-polymethylsiloxane). Helium was used as the carrier gas at a linear flow rate of 1.2 mL/min. The injection port temperature was maintained at 250°C. The oven temperature program was set to start at 40°C (held for 3.5 min), increased to 100°C at 10°C/min, then to 180°C at 7°C/min, and finally to 280°C at 25°C/min, holding for 5 min. Mass spectrometry detection was performed in electron impact ionization mode with an energy of 70 eV. The temperatures of the quadrupole mass detector, ion source, and transfer line were set to 150°C, 230°C, and 280°C, respectively. The mass spectrometry detection used selected ion monitoring mode for the identification and quantification of analytes.

#### 2.2.4 The animal care and model construction

Forty-eight healthy specific pathogen-free (SPF) Sprague-Dawley (SD) rats (3 weeks old, weighing 60 ± 10 g) were obtained from the Animal Experiment Center of Gansu University of Chinese Medicine. All experimental procedures were approved by the Animal Ethics Committee of Gansu University of Chinese Medicine.

The animals were randomly assigned using SPSS software to a blank control group (*n* = 8) and a model group (*n* = 40). Rats in the model group received daily intraperitoneal injections of 3,3′-iminodipropionitrile (IDPN; 300 mg/kg) for seven consecutive days to induce tic-like behaviors. On day 7, behavioral assessments were conducted based on predefined scoring criteria (Table 1). Rats with a total score ≥2 were considered successfully modeled. The blank control group received equal volumes of saline intraperitoneally for the same duration.

**TABLE 1:** Behavioral assessment score sheet.

Score	Motor behavior	Stereotyped behavior
0	Quiet or normal activity	No stereotyped behavior
1	Overexcited	Body rotation (clockwise or counterclockwise)
2	Increased exploratory behavior with discontinuous sniffing	Excessive up or down movement of the head and neck (an abnormal movement of the head and neck in the vertical direction to the ground)
3	Nonstop running	Excessive up or down movement of the head and neck plus rotation
4	Nonstop running accompanied by fearful jumping	Side-to-side head weaving, accompanied by excessive up or down movement of the head and neck

Following successful model establishment, the model group was further divided into five subgroups (*n* = 8 per group): model group, tiapride group, VOA group, SB203580 inhibitor group, and VOA + SB203580 combination group. The tiapride group was administered tiapride solution (3.194 mg/mL) via oral gavage. The VOA group received VOA at a dose of 51.12 mg/kg via gavage, a dosage determined based on behavioral assessments in a preliminary experiment. The SB203580 group received intraperitoneal injections of SB203580 (5 mg/kg). The combination group received both VOA (51.12 mg/kg, gavage) and SB203580 (5 mg/kg, intraperitoneal injection). The blank and model control groups received equal volumes of saline via gavage once daily for 28 days.

#### 2.2.5 Behavioral assessment

According to the behavioral scoring method (Fekete et al., 2021), rats were observed daily from 10:00 to 11:00 starting on the second day after modeling. Observations were conducted in a double-blind manner, recording scores every 5 min, and calculating the total and average scores. The scoring criteria are shown in Table 1.

#### 2.2.6 Sample collection and processing

Following the behavioral tests, rats from each group were anesthetized using 3% sodium pentobarbital (35 mg/kg) administered intraperitoneally. Four animals per group were randomly selected for transcardial perfusion. After thoracotomy, the heart was exposed and incised at the apex. A catheter was inserted into the left ventricle and advanced into the ascending aorta, followed by incision of the right atrium. The heart was perfused with 150–200 mL of saline until clear outflow from the right atrium was achieved. This was followed by continuous perfusion with 150 mL of 4% paraformaldehyde at 4°C (100 mL rapid perfusion for 2 min, followed by 50 mL slow perfusion). Brain tissue was then extracted and fixed overnight in 4% paraformaldehyde at 4°C, then immersed in 30% sucrose solution. The remaining four rats were used for cardiac blood collection. After centrifugation, plasma was collected and stored at −80°C for subsequent analysis. The rats were subsequently decapitated, and brain tissues were quickly dissected and separated into different regions, including the striatum. Tissue samples were placed into cryovials, flash-frozen in liquid nitrogen, and stored at −80°C until use.

#### 2.2.7 Nissl staining

The fixed biological tissue was finely trimmed to remove excess tissue and retain the area of interest. The trimmed tissue samples were dehydrated using gradient alcohol solutions (70%, 80%, 85%, 90%, 95%, 100%), each for 10 min. After dehydration, the tissue was immersed in xylene I and II for 25 min each for clearing. The cleared tissue was then immersed in melted paraffin for embedding preparation. Using an embedding machine (LeiCa, Germany), the tissue was embedded in paraffin to form tissue blocks. The blocks were sliced into approximately 4 μm thick sections using a microtome (LeiCa, Germany) and attached to pre-treated slides. The slides were baked in a drying oven (Shanghai Jinghong Experimental Equipment Co., China) at 65°C for 1 h to ensure complete paraffin solidification. The baked sections were deparaffinized and rehydrated through xylene (two steps, each for 25 min) and gradient ethanol (100%–70%, each for 10 min). The sections were washed twice with water. Nissl staining was performed using the Nissl staining kit (methyl violet method) for 15 min, followed by washing with tap water. Sections were differentiated for 5 s to optimize staining and washed again. The sections were then dehydrated again through gradient ethanol to xylene clearing. Finally, the sections were mounted with neutral balsam, avoiding bubbles. The mounted sections were observed under an inverted optical microscope (Olympus, Japan), and high-quality images were captured for analysis.

#### 2.2.8 Enzyme-linked immunosorbent assay (ELISA)

The experimental procedures include the preparation of standards and samples, ELISA steps, washing, color development, and absorbance measurement. Specifically, 50 μL of standards at different concentrations were added to the designated wells. Concurrently, blank wells and sample wells were set up. For the sample wells, 40 μL of sample diluent was added first, followed by 10 μL of the sample to achieve a final 5-fold dilution. Care was taken to avoid contact with the well walls, and the contents were gently mixed to ensure homogeneity. The plate was then incubated at 37°C for 30 min. Washing solution was prepared by diluting with distilled water at a ratio of 1:30 (1:20 for a 48-well plate). Following incubation, the plate was unsealed, and the contents were discarded. Each well was gently blotted to remove residual liquid. Washing solution was added to each well, allowed to sit for 30 s, and then discarded. This washing step was repeated five times. Subsequently, 50 μL of enzyme-labeled reagent was added to each well (except the blank wells) and incubated at 37°C for 30 min, followed by the same washing procedure. Next, 50 μL each of color reagents A and B were added to each well, gently mixed, and incubated in the dark at 37°C for 15 min. The reaction was terminated by adding 50 μL of stop solution, turning the solution from blue to yellow. Finally, the absorbance at 450 nm was measured within 15 min of adding the stop solution using a microplate reader (Thermo, United States), thus completing the ELISA detection process.

#### 2.2.9 Immunofluorescence

Fixed tissue samples were properly trimmed and dehydrated in a gradient of ethanol solutions based on tissue type and size. Tissues were cleared in xylene, embedded in paraffin using an embedding machine, and sectioned into approximately 4 μm thick slices using a microtome. The sections were gently spread in warm water, mounted on anti-off slides, and baked at 65°C for 1 h in a bench-top electric thermostatic drying oven, then stored at room temperature. The sections were deparaffinized with three changes of xylene (10 min each) and rehydrated in a graded series of ethanol (100%, 95%, 85%, 75%, each for 3 min) followed by gentle washing with running water and PBS immersion for 5 min. Antigen retrieval was performed by heating the sections in sodium citrate buffer for two 5-min cycles, followed by cooling to room temperature and three washes with PBS (5 min each). Endogenous peroxidase activity was blocked using 3% hydrogen peroxide for 15 min, followed by three PBS washes. The sections were incubated with 0.5% Triton X-100 for 15 min at room temperature to increase cell membrane permeability, followed by three PBS washes. Normal goat serum was applied to the sections for 15 min at room temperature to block nonspecific binding. Without washing, excess serum was blotted off, and the sections were incubated with primary antibodies (1:200 dilution) overnight at 4°C in a humid chamber. The next day, the sections were incubated at 37°C for 35 min, washed three times with PBS, and incubated with the corresponding fluorescent secondary antibody in the dark at 37°C for 60 min. After three PBS washes, the nuclei were stained with DAPI for 5 min, followed by three PBS washes. The sections were mounted with anti-fluorescence quenching mounting medium and immediately observed and photographed using an inverted fluorescence microscope (Olympus, Japan).

#### 2.2.10 Immunohistochemistry

Fixed tissue samples were trimmed and dehydrated based on tissue type and size using a dehydrator (LeiCa, Germany) with a gradient series of ethanol solutions. After dehydration, tissues were cleared in xylene and embedded in paraffin using an embedding machine. Paraffin-embedded tissues were sectioned into approximately 4 μm thick slices using a microtome. The sections were spread in warm water, mounted on anti-off slides, and baked at 65°C for 1 h in a bench-top electric thermostatic drying oven. After cooling to room temperature, slides were deparaffinized in xylene and rehydrated through a descending ethanol gradient, followed by rinsing under running water and immersion in PBS for 5 min. Heat-induced antigen retrieval was performed using sodium citrate buffer for two cycles of 5 min each, with cooling to room temperature and PBS washing. Endogenous peroxidase activity was blocked with 3% hydrogen peroxide for 15 min, followed by three PBS washes. Normal serum blocking was performed with goat serum from the rabbit SP kit at room temperature for 20 min. Primary antibodies (1:200 dilution) were applied, and sections were incubated overnight at 4°C in a humid chamber. The sections were then incubated at 37°C, washed, and incubated with biotinylated secondary antibody (yellow reagent) from the rabbit SP kit, followed by streptavidin-HRP (orange reagent) incubation. DAB staining was performed, and color development was stopped when a brown-yellow color appeared, standardizing the staining time for all sections. Sections were counterstained with hematoxylin, blued, dehydrated through graded alcohols, and cleared in xylene. Finally, sections were mounted with neutral balsam, avoiding air bubbles, and observed and photographed using an inverted optical microscope for further analysis.

#### 2.2.11 Quantitative real-time PCR (RT-qPCR)

Cells were harvested into centrifuge tubes and centrifuged at 12,000 rpm for 5 min at 4°C using a high-speed refrigerated centrifuge (BECKMAN, United States) to collect cell pellets. RNA was extracted using RNAiso Plus, and cells were thoroughly ground, then transferred to 1.5 mL RNase-free EP tubes and allowed to stand at room temperature for 5 min. The mixture was centrifuged at 12,000 rpm for 10 min at 4°C. The supernatant was carefully transferred to a new 1.5 mL EP tube, mixed with 0.2 mL chloroform, and vortexed for 15 s. After incubation at room temperature for 5 min, the sample was centrifuged at 12,000 rpm for 15 min at 4°C. The upper aqueous phase containing RNA was transferred to a new tube, mixed with 0.5 mL isopropanol, incubated at room temperature for 10 min, and then centrifuged at 12,000 rpm for 10 min at 4°C. The supernatant was discarded, and the RNA pellet was washed with 1 mL of 75% ethanol, centrifuged at 7,500 rpm for 5 min, air-dried, and dissolved in RNase-free water, then stored at −80°C. RNA concentration (ng/μL) and purity were assessed using a micro-spectrophotometer, ensuring an A260/A280 ratio between 1.8 and 2.1. Reverse transcription was performed in a microcentrifuge tube using 8×gDNA Remover and RNase-free ddH_2_O, with a final reaction volume of 16 μL. The reaction mixture included 5×RT SuperMix and was gently mixed. The reverse transcription conditions were set at 37°C for 15 min, followed by enzyme inactivation at 85°C for 5 s. The resulting cDNA was used for PCR amplification in a two-step process with 20 μL reaction volume per template in triplicate. The steps included 95°C for 30 s for pre-denaturation, followed by 40 cycles of 95°C for 10 s and 60°C for 30 s for annealing and extension. Fluorescence changes were monitored using a real-time PCR instrument (Bio-Rad, United States) with data analysis performed. The sequences of all RT-qPCR primers used in this study are listed in Table 2.

**TABLE 2:** Shows the sequences of all RT-qPCR primers used.

Primers	Forward sequence (5′–3′)	Reverse sequence (5′–3′)	Length (bp)
GAPDH	TGA​TGG​GTG​TGA​ACC​ACG​AG	AGT​GAT​GGC​ATG​GAC​TGT​GG	152
NLRP3	CTC​GCA​TTG​GTT​CTG​AGC​TC	AGT​AAG​GCC​GGA​ATT​CAC​CA	153
p38 MAPK	ACC​CTT​GCC​ATC​TGA​CAT​CA	TCC​CTT​TGT​TCG​GTT​TGC​AG	145
STAT3	GAA​CTG​AGT​GAG​CGT​GGG​TGA​TG	AGG​ACA​GGC​GGA​CAG​AAC​ATA​GG	128

#### 2.2.12 Western blot

RIPA lysis buffer containing PMSF was prepared and stored at −20°C. Frozen tissue samples (100 mg) were thawed, minced with sterilized scissors, and lysed on ice with RIPA buffer containing PMSF, periodically adding PMSF to prevent protein degradation. The lysate was transferred to 2 mL centrifuge tubes and centrifuged at 12,000 rpm for 15 min at 4°C using a high-speed refrigerated centrifuge. The supernatant was collected, and protein concentration was determined using the BCA protein assay kit with a standard curve method, measuring absorbance at 562 nm with a microplate reader. Protein samples were mixed with 5×loading buffer, denatured at 99°C in a metal bath, rapidly cooled, and stored at −80°C. Glass plates were cleaned, and 10% resolving gel and 5% stacking gel were prepared for electrophoresis (120 V). The gel and PVDF membrane (pre-activated in methanol) were equilibrated in transfer buffer, and the “sandwich” was assembled for transfer (400 mA for 90 min). The PVDF membrane was blocked with 5% non-fat milk prepared in TBST for 1 h at room temperature, then incubated overnight at 4°C with primary antibodies against COX-2, CD11b, and CD163 (all diluted 1:1,000). The membrane was washed with TBST, incubated with goat anti-rabbit secondary antibody (1:4,000 dilution) at room temperature for 2 h, and washed again. Enhanced chemiluminescence (ECL) substrate was applied to the membrane, and protein bands were visualized using an X-ray film cassette (Shantou Yongtai Medical Equipment Co., China). The bands were analyzed using ImageJ software to determine the relative expression of target proteins.

#### 2.2.13 Statistical analysis

Statistical analysis was performed using SPSS 26.0 software, and data visualization was done using GraphPad Prism 9.0 software. All quantitative data were expressed as means ± standard deviation (SD). Group comparisons were made using one-way ANOVA followed by LSD and Dunnett’s *post hoc* tests for multiple comparisons. Repeated measures ANOVA was used for comparisons at different time points. *P* < 0.05 was considered statistically significant.

## 3 Results

### 3.1 Identification of volatile metabolites by GC-MS

**FIGURE 1:**
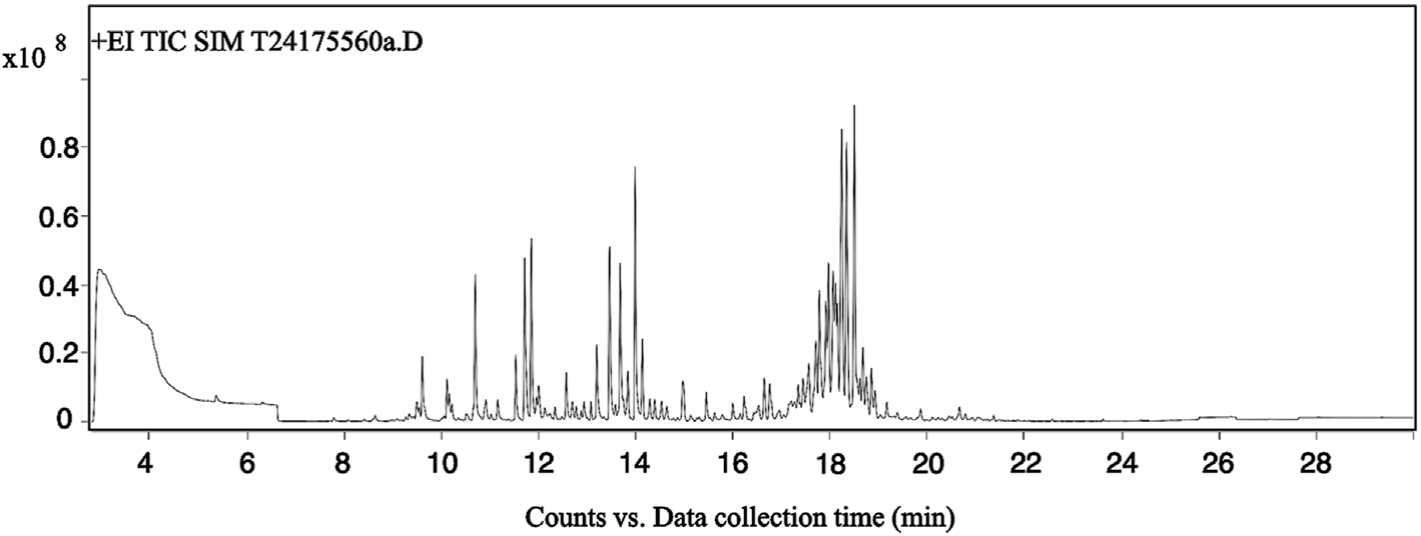
Total ion chromatogram of VOA analyzed by gas chromatography-mass spectrometry.

The total ion chromatogram (TIC) of *Acorus tatarinowii* volatile oil obtained by gas chromatography-mass spectrometry (GC-MS) is shown in Figure 1. Through GC-MS analysis, a total of 1,624 metabolites were identified in the volatile oil of *A. tatarinowii*, primarily including terpenes, esters, ketones, alcohols, and ethers. The 15 most prominent metabolites are listed in Table 3. The identification of these metabolites is significant for understanding the chemical composition and biological activity of *A. tatarinowii* volatile oil.

**TABLE 3:** Partial Chemical metabolites of VOA.

No.	Metabolite	RT (min)	Formula	CAS	Peak area (%)
1	3-Methyl-pyrrolo (2,3-b)pyrazine	18.25	C7H7N3	20321-99-7	2.52
2	.alpha.-Irone	18.33	C14H22O	79-69-6	2.38
3	1-Phenylcyclohexylamine	18.25	C12H17N	2201-24-3	2.32
4	.beta.-Asarone	20.79	C12H16O3	5273-86-9	2.11
5	Citral	13.99	C10H16O	5392-40-5	1.76
6	(1R,5S)-1,8-Dimethyl-4-(propan-2-ylidene)spiro [4.5]dec-7-ene	18.34	C15H24	28400-12-6	1.39
7	(3aS,8aS)-6,8a-Dimethyl-3-(propan-2-ylidene)-1,2,3,3a,4,5,8,8a-octahydroazulene	18.34	C15H24	395070-76-5	1.39
8	Geraniol	13.68	C10H18O	106-24-1	0.95
9	Benzene,1-methyl-4-(1,2,2-trimethylcyclopentyl)-, (R)-	18.25	C15H22	16982-00-6	0.91
10	Naphthalene,1,2,3,4-tetrahydro-1,6-dimethyl-4-(1-methylethyl)-, (1S-cis)-	18.51	C15H22	483-77-2	0.89
11	trans-Calamenene	18.51	C15H22	73209-42-4	0.89
12	cis-Calamenene	18.51	C15H22	72937-55-4	0.89
13	3,5,11-Eudesmatriene	18.25	C15H22	193615-07-5	0.84
14	Eudesma-2,4,11-triene	18.25	C15H22	82462-31-5	0.84
15	(2R,8R,8aS)-8,8a-Dimethyl-2-(prop-1-en-2-yl)-1,2,3,7,8,8a-hexahydronaphthalene	18.25	C15H22	5090-61-9	0.84

### 3.2 VOA effectively improves motor and stereotypic behaviors in TS rats

**FIGURE 2:**
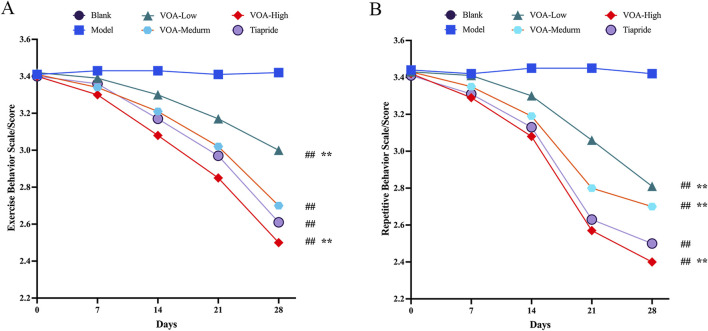
All dosage groups of VOA (High, Medium, and Low) as well as the Tiapride group significantly reduced motor and stereotypic behaviors in rats, with the High-dose VOA group demonstrating superior therapeutic effects compared to the Tiapride group. **(A)** Line graph depicting changes in motor behavior in each group at 0, 7, 14, 21 and 28 days post-modeling. **(B)** Line graph depicting changes in stereotypic behavior in each group at 0, 7, 14, 21 and 28 days post-modeling. Data are presented as means ± standard deviation (*n* = 8). ^##^ *P* < 0.01 compared with the Model group; ^*^ *P* < 0.05, ^**^ *P* < 0.01 compared with the Tiapride group.

This study assessed changes in motor and stereotypic behaviors in rats before and after drug intervention among the Blank group, Model group, Low-dose VOA group (12.78 mg/kg), Medium-dose VOA group (25.6 mg/kg), High-dose VOA group (51.12 mg/kg), and Tiapride group (48 mg/kg) (*n* = 8). The results, shown in Table 4, Table 5 and Figure 2, indicate that, compared to the Model group, rats in the Low-, Medium-, and High-dose VOA groups, as well as the Tiapride group, exhibited significantly lower scores in motor and stereotypic behaviors after intervention (*P* < 0.01). The High-dose VOA group showed lower scores in both behaviors compared to the Tiapride group (*P* < 0.01), while the Low- and Medium-dose VOA groups had higher scores than the Tiapride group (*P* < 0.05). Additionally, in our previous studies (Feng et al., 2024), we first evaluated the therapeutic effects of different doses of VOA on a TS rat model, and the results demonstrated that High-dose VOA exhibited significant anti-tic effects. These findings suggest that the High-dose VOA group outperforms Tiapride in improving both behavior scores, highlighting VOA’s potential as a therapeutic agent for TS. Consequently, High-dose VOA (51.12 mg/kg) was chosen for subsequent experiments.

**TABLE 4:** Comparison of motor behavior scores among different groups of rats.

Group	*n*	Pre-intervention^a^	Day 7^a^	Day 14^a^	Day 21^a^	Day 28^a^
Blank group	8	0.00 ± 0.00	0.00 ± 0.00	0.00 ± 0.00	0.00 ± 0.00	0.00 ± 0.00
Model group	8	3.41 ± 0.14	3.43 ± 0.16	3.43 ± 0.19	3.41 ± 0.11	3.42 ± 0.10
Low-dose VOA group	8	3.42 ± 0.32	3.39 ± 0.15	3.30 ± 0.13	3.17 ± 0.13	3.00 ± 0.12^##■■^
Medium-dose VOA group	8	3.41 ± 0.14	3.34 ± 0.05	3.21 ± 0.13^##^	3.02 ± 0.12^##▲^	2.70 ± 0.12^##▲▲^
High-dose VOA group	8	3.40 ± 0.16	3.30 ± 0.15^#^	3.08 ± 0.06^##▲^	2.85 ± 0.10^##▲▲□^	2.55 ± 0.15^##▲▲□□■^
Tiapride group	8	3.40 ± 0.12	3.36 ± 0.11	3.17 ± 0.23^##^	2.97 ± 0.23^##▲▲^	2.61 ± 0.15^##▲▲^

^a^ The values represent the means ± standard deviation of stereotypic behavior scores before drug intervention and on days 7, 14, 21, and 28 post-intervention.

^#^ *P* < 0.05.

^##^ *P* < 0.01 compared with the Model group;

^▲^ *P* < 0.05.

^▲▲^ *P* < 0.01 compared with the Low-dose VOA, group.

^□^ *P* < 0.05.

^□□^ *P* < 0.01 compared with the Medium-dose VOA, group.

^■^ *P* < 0.05.

^■■^ *P* < 0.01 compared with the Tiapride group.

**TABLE 5:** Comparison of stereotypic behavior scores among different groups of rats.

Group	*n*	Pre-intervention^a^	Day 7^a^	Day 14^a^	Day 21^a^	Day 28^a^
Blank group	8	0.00 ± 0.00	0.00 ± 0.00	0.00 ± 0.00	0.00 ± 0.00	0.00 ± 0.00
Model group	8	3.44 ± 0.11	3.42 ± 0.11	3.45 ± 0.09	3.45 ± 0.10	3.42 ± 0.11
Low-dose VOA group	8	3.43 ± 0.09	3.41 ± 0.05	3.30 ± 0.09^##^	3.06 ± 0.11^##^	2.81 ± 0.12^##■■^
Medium-dose VOA group	8	3.43 ± 0.12	3.35 ± 0.06	3.19 ± 0.12^##▲^	2.80 ± 0.09^##▲▲^	2.70 ± 0.14^##▲■■^
High-dose VOA group	8	3.42 ± 0.09	3.29 ± 0.11^##▲▲^	3.11 ± 0.04^##▲▲^	2.60 ± 0.12^##▲▲□□^	2.48 ± 0.10^##▲▲□□■^
Tiapride group	8	3.41 ± 0.08	3.31 ± 0.11^#▲^	3.13 ± 0.19^##▲▲^	2.63 ± 0.08^##▲▲□□^	2.50 ± 0.06^##▲▲□□^

^a^ The values represent the means ± standard deviation of stereotypic behavior scores before drug intervention and on days 7, 14, 21, and 28 post-intervention.

^#^ *P* < 0.05.

^##^ *P* < 0.01 compared with the Model group;

^▲^ *P* < 0.05.

^▲▲^ *P* < 0.01 compared with the Low-dose VOA, group.

^□□^ *P* < 0.01 compared with the Medium-dose VOA, group.

^■^ *P* < 0.05.

^■■^ *P* < 0.01 compared with the Tiapride group.

### 3.3 VOA effectively mitigates neuronal damage in the striatum of TS rats

**FIGURE 3:**
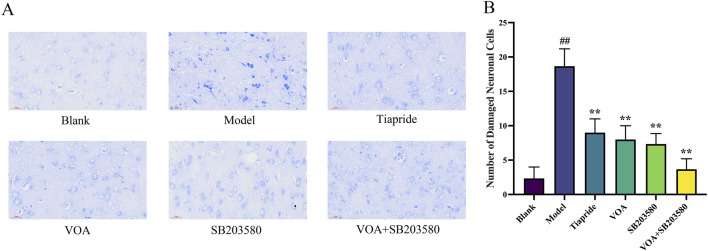
VOA repairs neuronal cells in the striatum of TS model rats. **(A)** Nissl staining compared neuronal structures in the striatum of TS rats among the Blank, Model, Tiapride, VOA, SB203580, and VOA + SB203580 groups (scale bar = 30 µm). All treatment groups showed improvements in the number and morphology of neurons compared to the Model group, with VOA and Tiapride exhibiting significant effects in promoting neuronal structure recovery. **(B)** Bar graph analysis of the number of damaged neurons. Data are presented as means ± standard deviation (*n* = 3). ^##^ *P* < 0.01 compared with the Blank group; ^**^ *P* < 0.01 compared with the Model group.

As shown in Figure 3, neurons in the Blank group exhibited intact structures, with evenly distributed and clearly defined Nissl bodies, indicating normal neuronal health. In the Model group, neurons in the striatum of TS rats displayed significant vacuolation and deformation, a reduction in the number of viable neurons, and blurred or absent Nissl bodies, indicating successful TS model construction with marked neuronal damage and inflammation. Neuronal morphology significantly improved in the VOA, Tiapride, SB203580, and VOA + SB203580 groups, with an increased number of viable neurons, more regular cell shapes, and better consistency in size, suggesting these treatments offer protective and reparative effects on neuronal damage. Particularly, Compared with the Model group, the number of damaged neurons was significantly reduced in all treatment groups (*P* < 0.01), with VOA showing a statistically significant effect in reducing neuronal damage. Notably, the neuronal structure in the VOA + SB203580 group most closely resembled the healthy state of the Blank group. Additionally, VOA demonstrated a slightly more significant effect than Tiapride in promoting neuronal recovery, potentially offering greater benefits in maintaining neuronal integrity and function.

### 3.4 VOA reduces pro-inflammatory cytokines IL-6 and TNF-α and increases anti-inflammatory cytokine IL-10 in TS rats

**FIGURE 4:**
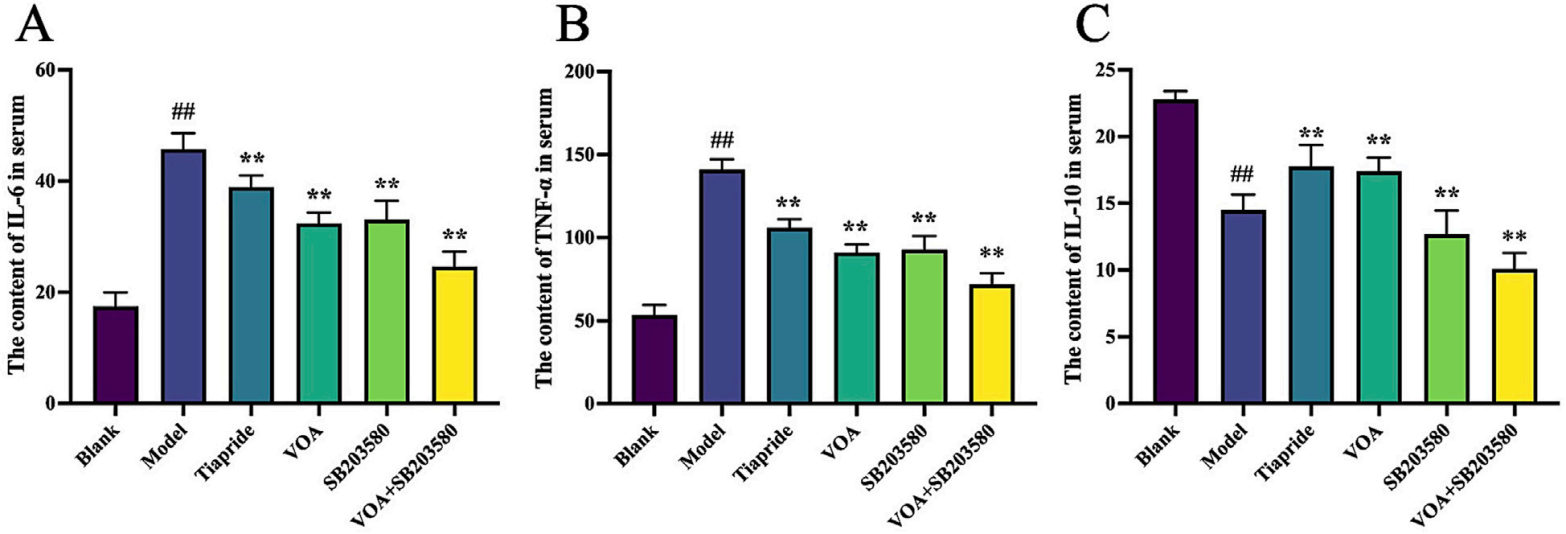
VOA regulates the overall inflammatory state by reducing pro-inflammatory cytokines and increasing anti-inflammatory cytokines in TS rats. **(A–C)** Quantitative analysis of inflammatory cytokine levels using ELISA. Data are presented as means ± standard deviation (*n* = 6). ^##^ *P* < 0.01 compared with the Blank group; ^**^ *P* < 0.01 compared with the Model group.

To assess whether VOA repairs neuroinflammation in TS rats by modulating inflammatory cytokines, we used ELISA to measure the levels of IL-6, TNF-α, and IL-10. Figures 4A–C show that, compared to the Model group, the VOA group reduced levels of IL-6 and TNF-α (*P* < 0.01) and increased levels of IL-10 (*P* < 0.01) in TS rats. Overall, VOA effectively lowered pro-inflammatory cytokines IL-6 and TNF-α and elevated the anti-inflammatory cytokine IL-10, indicating VOA’s efficacy in suppressing inflammatory responses in TS rats.

### 3.5 VOA inhibits CD11 b and COX-2 protein expression and increases CD163 protein expression in the striatum of TS rats

**FIGURE 5:**
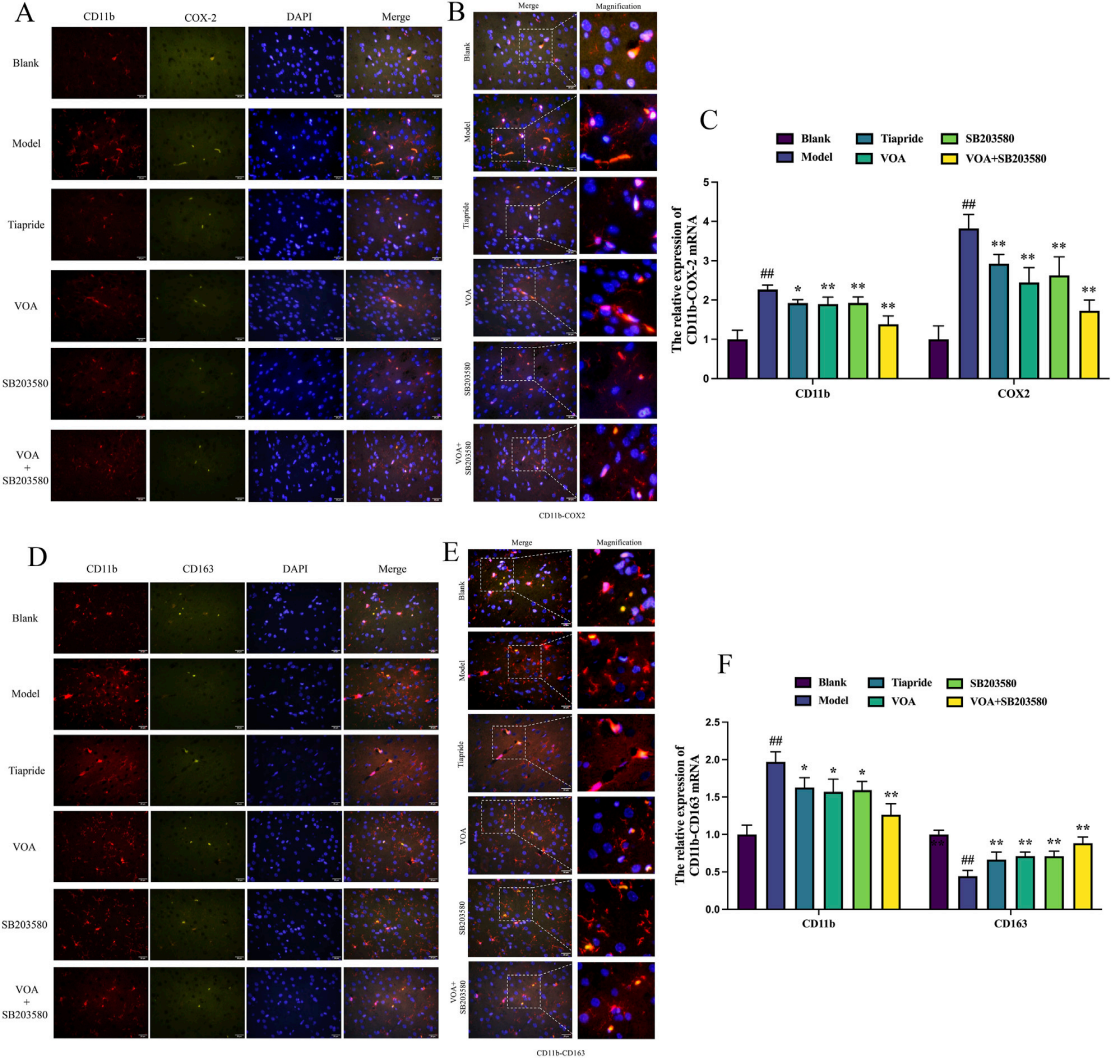
VOA inhibits CD11 b and COX-2 protein expression and increases CD163 protein expression in the striatum of TS rats. **(A, D)** Representative fluorescence images of CD11 b (red), COX-2 (green), CD163 (green), and DAPI (blue) (scale bar = 50 µm). **(B, E)** Enlarged representative merged fluorescence images (scale bar = 50 µm). **(C, F)** Bar chart analysis of CD11b, COX-2, and CD163 protein expression. Data are presented as means ± standard deviation (*n* = 3). ^##^ *P* < 0.01 compared with the Blank group; ^*^ *P* < 0.05, ^**^ *P* < 0.01 compared with the Model group.

Detecting the expression of CD11b, COX-2, and CD163 markers is crucial in neuroinflammation. We used immunofluorescence to measure the expression levels of M1 markers (CD11b, COX-2) and M2 markers (CD163) in the striatum of TS rats. Figures 5A–F show that, compared to the Model group, VOA reduced the expression levels of CD11 b and COX-2 proteins (*P* < 0.01) and increased the expression level of CD163 protein (*P* < 0.01). Studies have shown that CD163 can bind to its ligand to transduce signals, releasing anti-inflammatory mediators such as IL-10. IL-10 can further upregulate CD163 expression through autocrine or paracrine pathways, forming a positive feedback loop (Han et al., 2022). This positive loop aligns with the increased IL-10 expression in Figure 3C, promoting the polarization of microglia to an anti-inflammatory phenotype and regulating neuroinflammation in TS model rats.

### 3.6 VOA reduces NLRP3, Caspase-1, and GSDMD protein expression in microglia of TS rats

**FIGURE 6:**
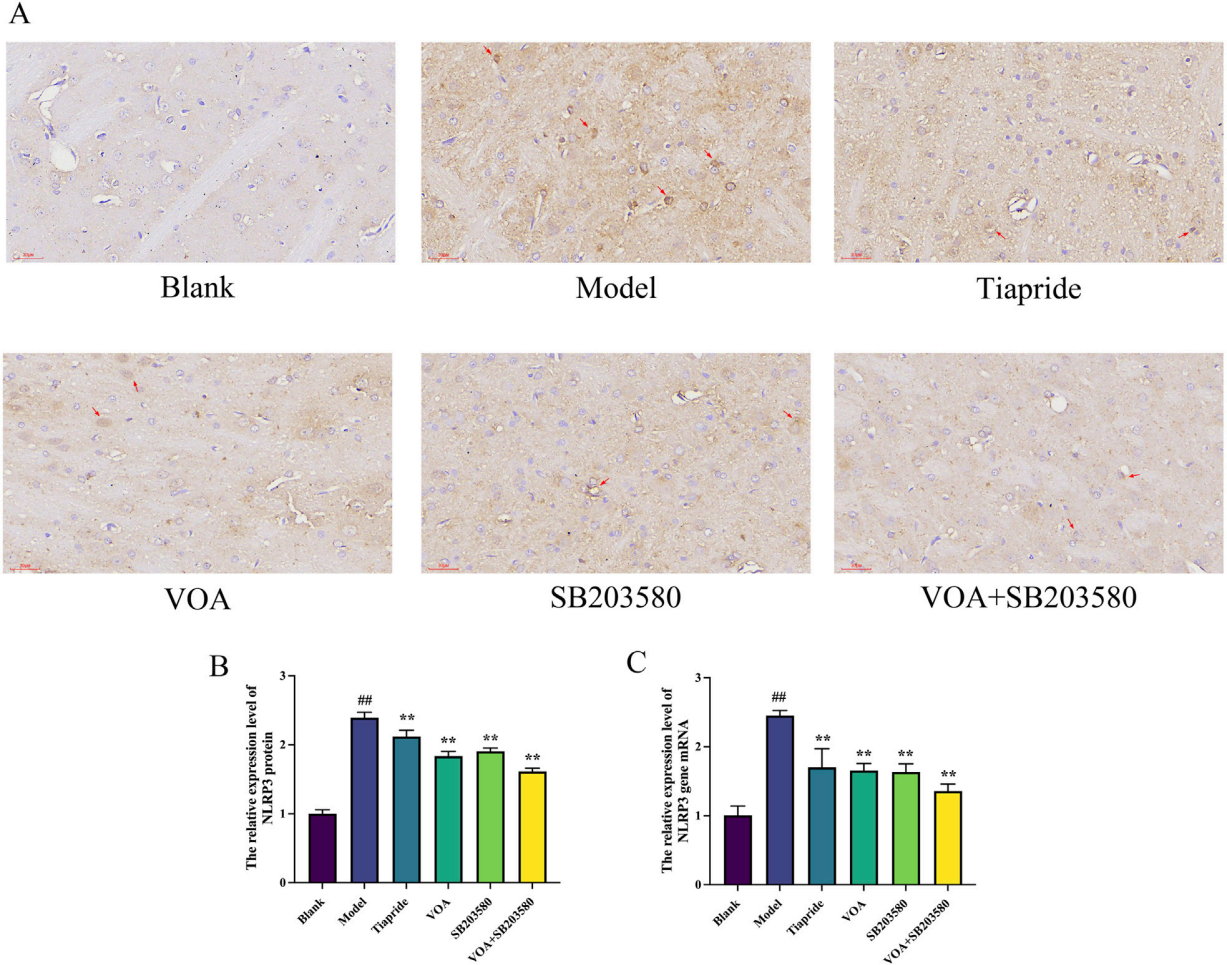
VOA inhibits NLRP3 protein and gene mRNA expression levels in the striatum of TS rats. **(A)** Immunohistochemistry images of NLRP3 protein (scale bar = 30 µm); **(B)** Bar chart showing relative expression levels of NLRP3 protein; **(C)** Bar chart showing relative expression levels of NLRP3 gene mRNA. Data are presented as means ± standard deviation (*n* = 3). ^##^ *P* < 0.01 compared with the Blank group; ^**^ *P* < 0.01 compared with the Model group.

**FIGURE 7:**
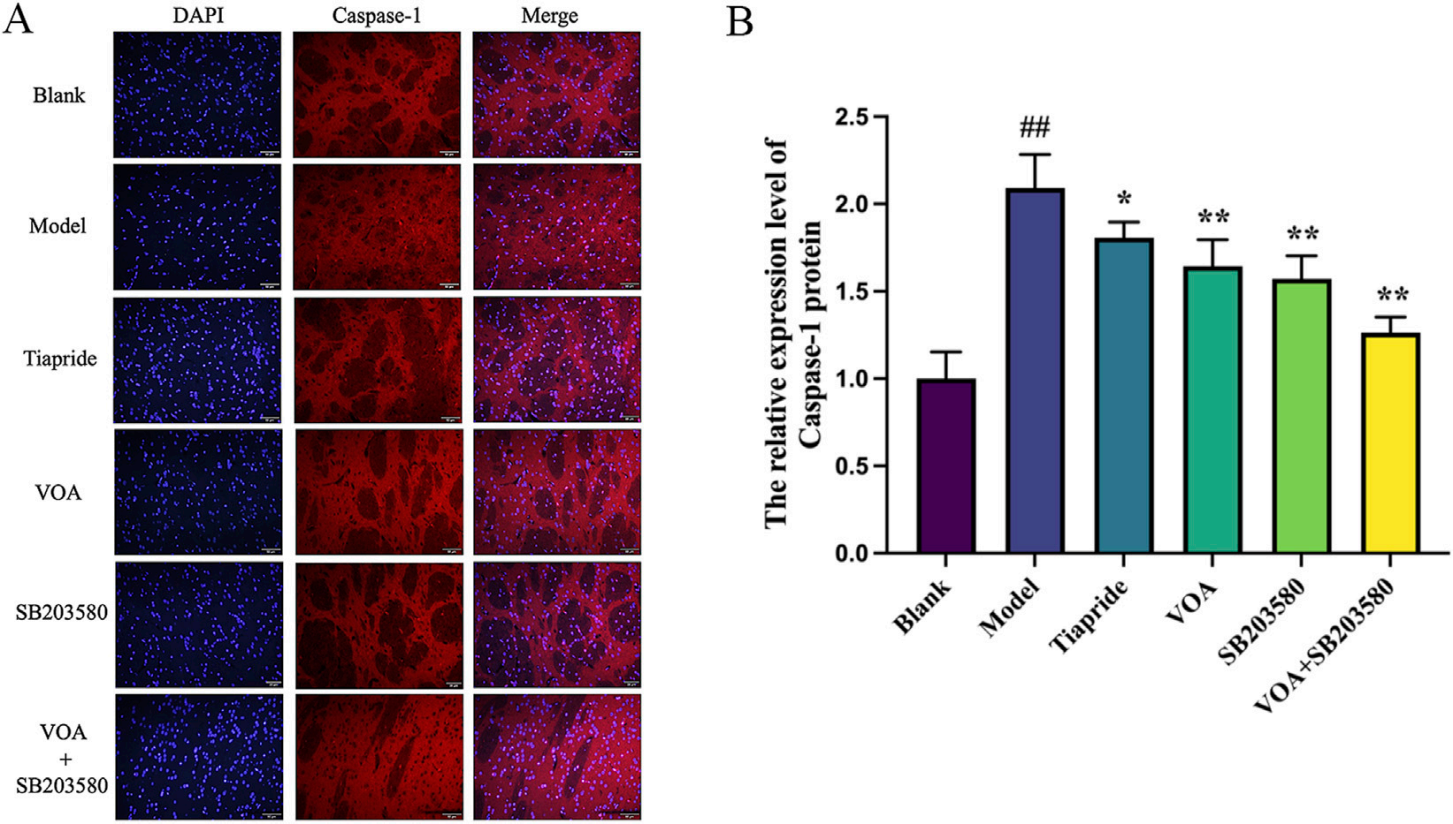
VOA reduces Caspase-1 protein expression levels in the striatum of TS rats. **(A)** Immunofluorescence images of Caspase-1 (scale bar = 50 µm); **(B)** Bar chart showing Caspase-1 protein expression levels. Data are presented as means ± standard deviation (*n* = 3). ^##^ *P* < 0.01 compared with the Blank group; ^*^ *P* < 0.05, ^**^ *P* < 0.01 compared with the Model group.

**FIGURE 8:**
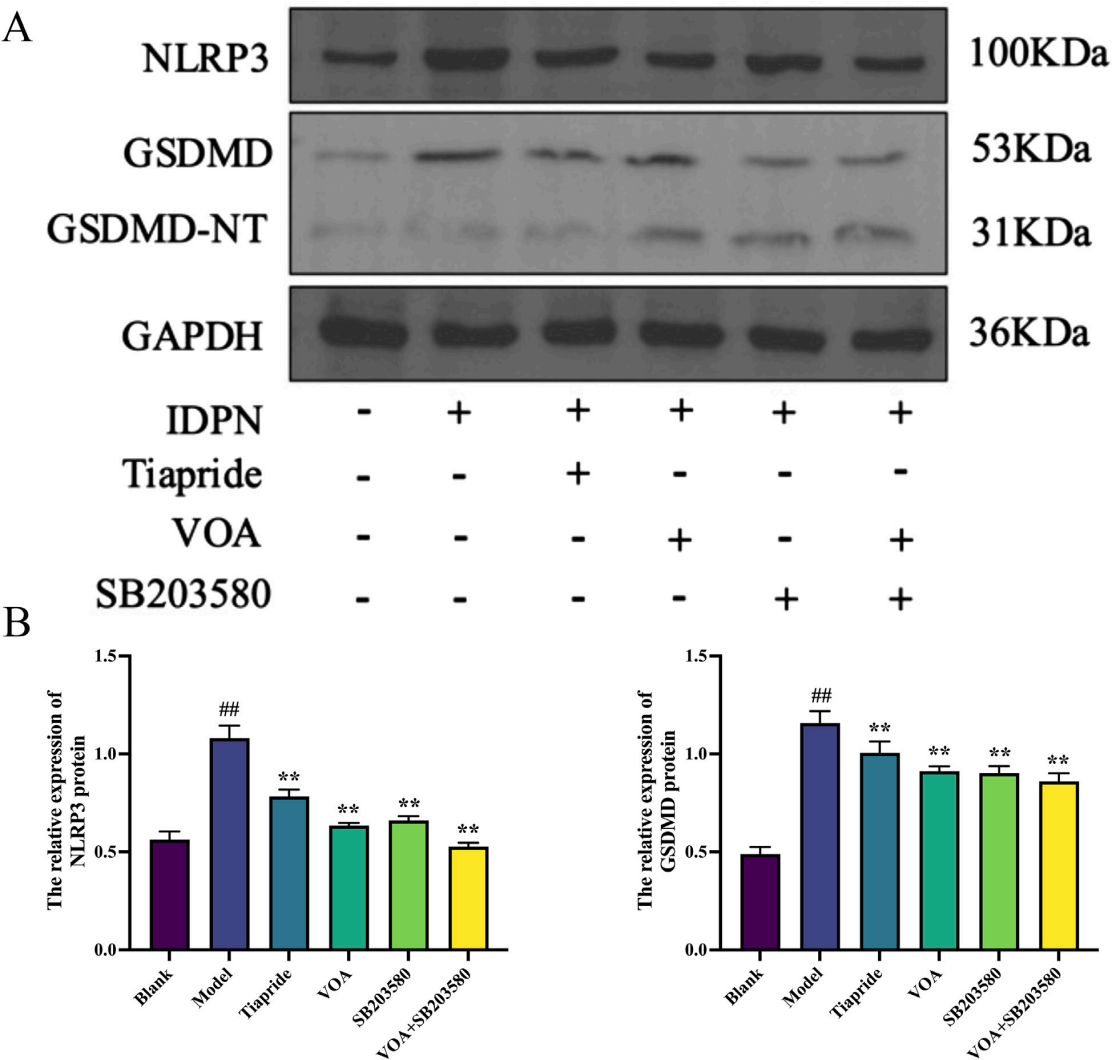
VOA inhibits NLRP3, GSDMD/GSDMD-NT protein expression levels in the striatum of TS rats. **(A)** Western blot images of NLRP3, GSDMD/GSDMD-NT proteins; **(B)** Bar chart showing relative expression levels of NLRP3, GSDMD proteins. Data are presented as means ± standard deviation (*n* = 3). ^##^ *P* < 0.01 compared with the Blank group; ^**^ *P* < 0.01 compared with the Model group.

Activation of the NLRP3 inflammasome is a key mediator in the release of inflammatory cytokines from microglia, playing a crucial role in microglial overactivation by activating its core metabolite Caspase-1 to enhance the inflammatory response (Gu et al., 2022; Yao et al., 2022). Using immunohistochemistry and RT-qPCR, we found that, Figures 6A–C show that, compared to the Model group, VOA and Tiapride treatment reduced NLRP3 gene mRNA and protein expression levels in the striatum of TS rats (*P* < 0.01). Subsequent Western blot analysis confirmed that VOA and Tiapride significantly downregulated NLRP3 protein expression levels (*P* < 0.01). Furthermore, Figure 7 show that, immunofluorescence analysis of Caspase-1 revealed that VOA and Tiapride significantly downregulated Caspase-1 protein expression levels compared to the Model group (*P* < 0.01). As shown in Figure 8, Western blot analysis of GSDMD showed that, compared to the Model group, VOA and Tiapride inhibited GSDMD protein expression levels (*P* < 0.01). These data suggest that VOA inhibits NLRP3 activation, reduces Caspase-1 activation, blocks GSDMD activation, decreases the production and release of pro-inflammatory cytokines, and protects neurons from inflammation-induced damage.

### 3.7 VOA reduces the activity of the p38 MAPK pathway and the phosphorylation levels of nuclear transcription factor STAT3

**FIGURE 9:**
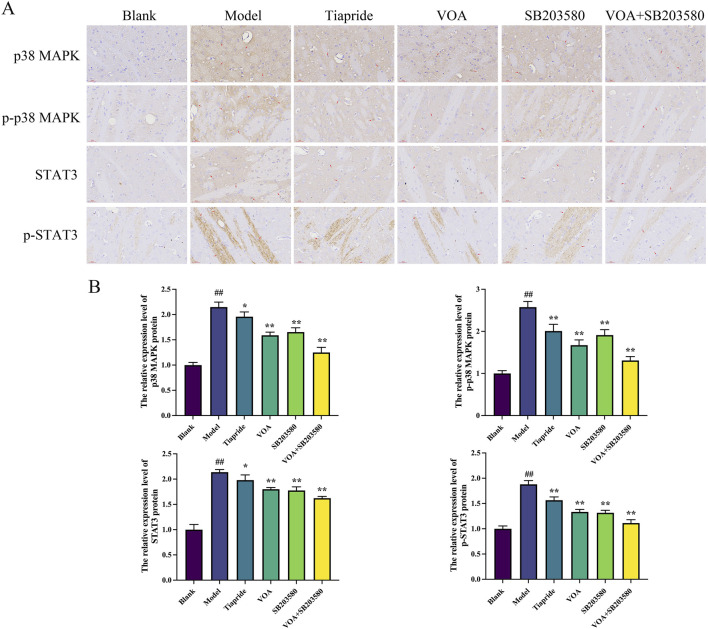
VOA reduces the expression of the p38 MAPK pathway and the phosphorylation levels of nuclear transcription factor STAT3. **(A)** Immunohistochemical images of p-p38 MAPK, p38 MAPK, p-STAT3, and STAT3 proteins (scale bar = 30 µm). **(B)** Bar graphs depicting the relative expression levels of p-p38 MAPK, p38 MAPK, p-STAT3, and STAT3 proteins. Data are presented as means ± standard deviation (*n* = 3). ^##^ *P* < 0.01 compared to the Blank group; ^*^ *P* < 0.05, ^**^ *P* < 0.01 compared to the Model group.

**FIGURE 10:**
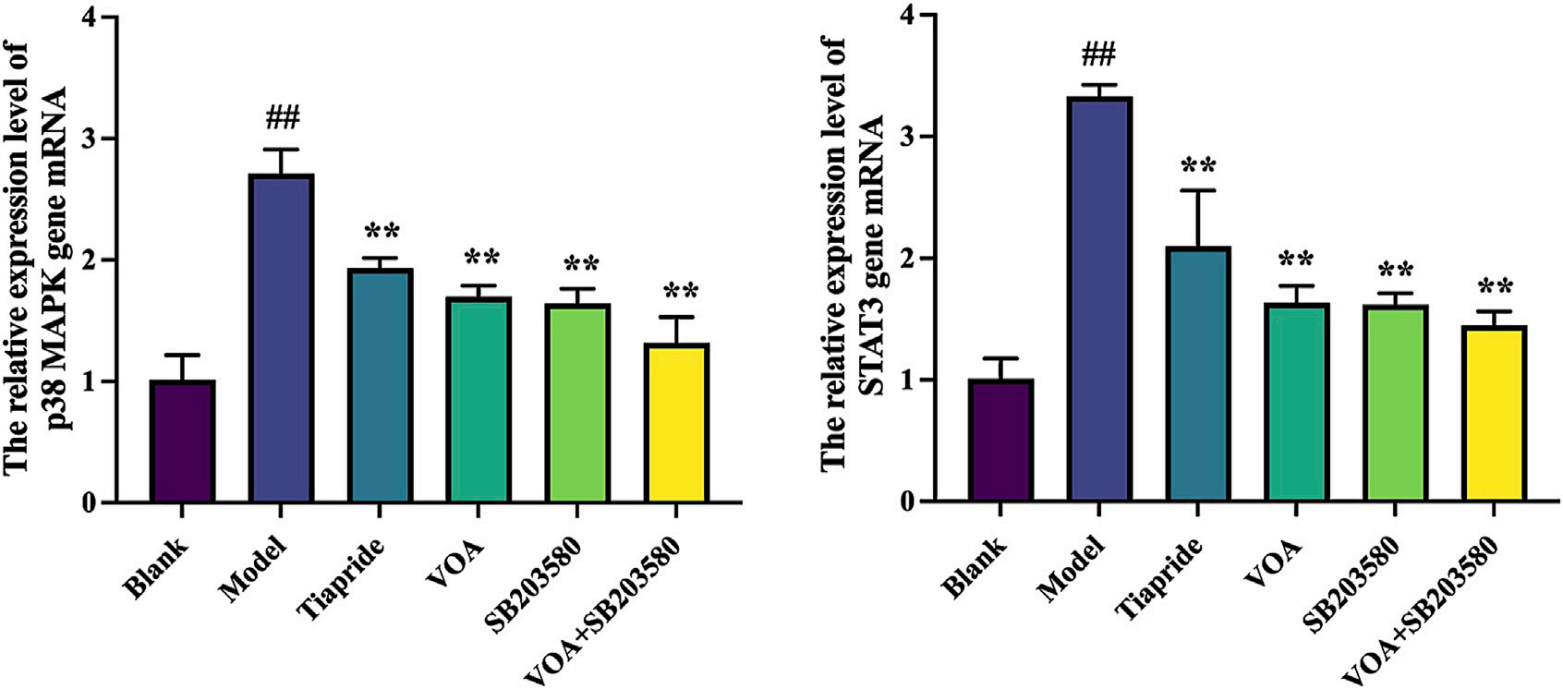
VOA reduces the mRNA expression levels of p38 MAPK and STAT3 genes. Bar graphs show the relative expression levels of p38 MAPK and STAT3 gene mRNA. Data are presented as means ± standard deviation (*n* = 3). ^##^ *P* < 0.01 compared to the Blank group; ^**^ *P* < 0.01 compared to the Model group.

**FIGURE 11:**
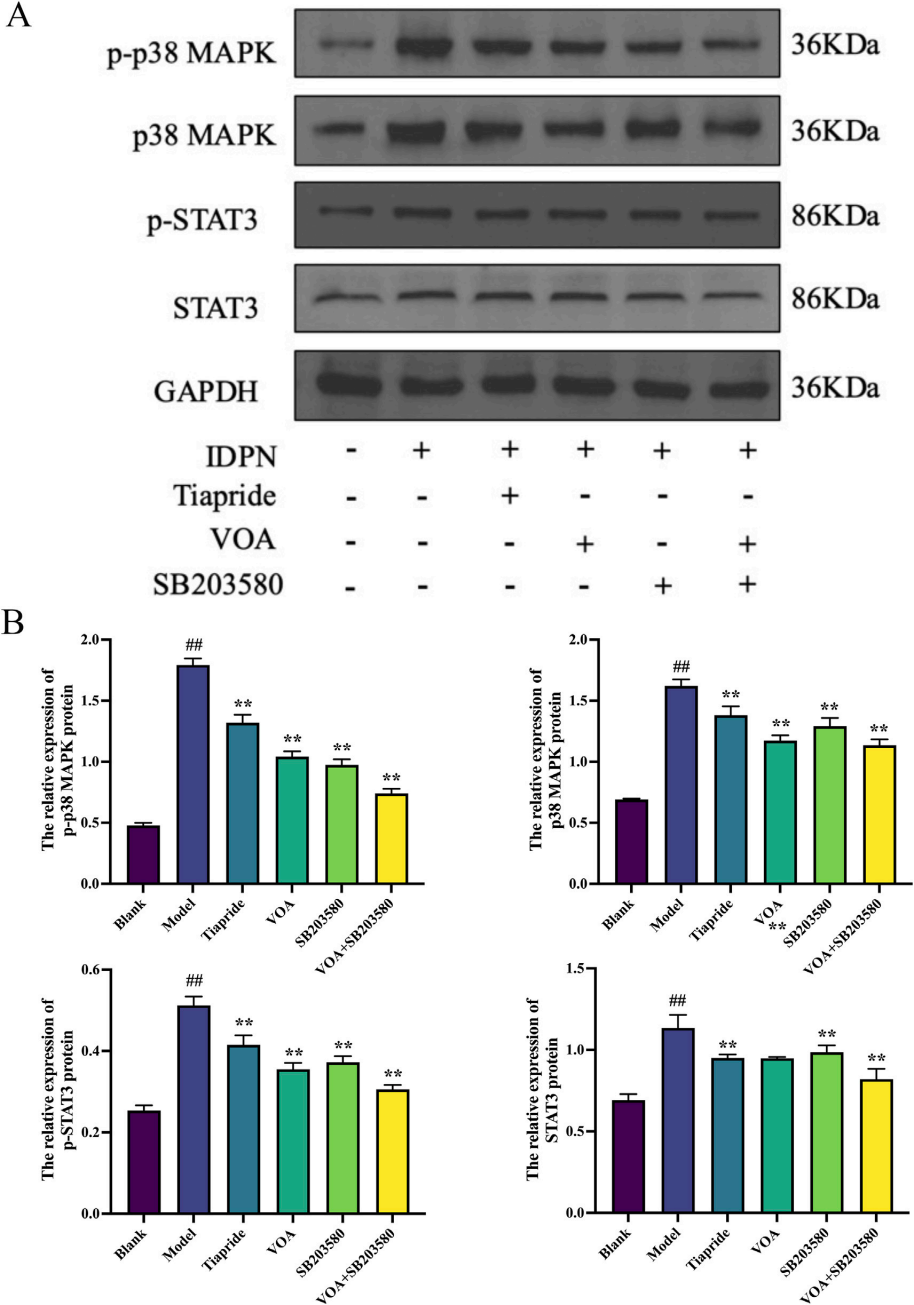
VOA reduces the expression levels of p-p38 MAPK, p38 MAPK, p-STAT3, and STAT3 proteins. **(A)** Western blot images of p-p 38MAPK, p38 MAPK, p-STAT3, and STAT3 proteins. **(B)** Bar graphs showing the relative expression levels of p-p38 MAPK, p38 MAPK, p-STAT3, and STAT3 proteins. Data are presented as means ± standard deviation (*n* = 3). ^##^ *P* < 0.01 compared to the Blank group; ^**^ *P* < 0.01 compared to the Model group.

p38 MAPK is a critical signaling pathway involved in the activation of neuroinflammatory responses (Hu et al., 2022), and STAT3 serves as a common intracellular signaling route among various inflammatory cells and mediators (Huang et al., 2021). To verify the trend of neuroinflammatory response in this study, we employed immunohistochemical analysis using anti-p-p38 MAPK and anti-p-STAT3 antibodies. As shown in Figures 9A,B, VOA significantly reduced the protein expression levels of p-p38 MAPK and p-STAT3 compared to the Model group (*P* < 0.01). Subsequent reverse transcription polymerase chain reaction (RT-qPCR) analysis, illustrated in Figure 10, further demonstrated that VOA markedly downregulated the expression levels of p38 MAPK and STAT3 proteins (*P* < 0.01). Moreover, Western blot analysis following VOA treatment, presented in Figures 11A,B, corroborated these findings by confirming that VOA effectively decreased the expression levels of p38 MAPK and STAT3 proteins (*P* < 0.01). The trend observed in the Inhibitor SB203580 group further validated the activation state and the potential for intervention in these specific signaling pathways in the pathogenesis of TS. Through comprehensive analyses involving immunohistochemistry, RT-qPCR, and Western blot, these results collectively suggest that VOA modulates neuroinflammation in the striatum of TS rats by regulating the p38 MAPK/STAT3 signaling pathway.

## 4 Discussion

As previously mentioned, TD are a group of disorders that can be classified into PTD, CTD and TS based on the duration and type of symptoms. TS falls within the spectrum of TD and represents the most severe form. TS and TD share common characteristics, including involuntary, sudden, rapid, and repetitive movements or vocalizations (Liu et al., 2020). While distinguishing between these disorders aids clinical diagnosis, it can be challenging in clinical practice to accurately differentiate between TS and TD in a short period of time. When patients present with severe symptoms and it is difficult to immediately distinguish between TS and TD, the primary goal of treatment is to alleviate symptoms and improve the patient’s quality of life. Medications that can treat both types of tics are more aligned with clinical needs. Additionally, TD and TS share many pathological similarities, including neurotransmitter imbalances (such as dopamine) and abnormalities in the cortico-basal ganglia-thalamic circuitry (Jindachomthong et al., 2022; Ricketts, 2023). Neuroinflammation plays a crucial role in the pathogenesis of both TS and TD, with excessive activation of inflammatory factors potentially affecting the function of neural networks in the basal ganglia, leading to or exacerbating tic symptoms (Almeida et al., 2015; Skarphedinsson et al., 2015; Ramkiran et al., 2019). Thus, many medications used in clinical practice can treat both conditions simultaneously. VOA mentioned in this study is similarly applicable to both tic disorders, and based on our previous research, we further explored the efficacy of VOA in treating TS. This study focused on the role of the p38 MAPK/NLRP3/STAT3 signaling pathway in TS and demonstrated that VOA can inhibit pro-inflammatory cytokines, reduce microglial activation, and regulate neuroinflammation, thereby showing its potential efficacy in treating TS.

In our previous research (Feng et al., 2024), we found that VOA has a significant therapeutic effect on TD, with the highest efficacy observed in the High-dose group (51.12 mg/kg). TS is the most common manifestation of TD. IDPN is a commonly used neurotoxin. The IDPN model was first utilized by Diamond et al. (1982) to develop the TD model and can significantly increase stereotypic and motor behavior scores in rats (Cherry et al., 2014; Diamond et al., 1982). The IDPN model is a standard animal model for TS, capable of comprehensively reproducing the behavioral characteristics of TS; it has been used in many TS experiments (Zhao et al., 2015; Liu et al., 2020; Wang N. et al., 2022; Yang et al., 2022) and activate microglia in the striatum of these rats (Zhao et al., 2020; Wang X. et al., 2023). Therefore, in this study, we used an IDPN-induced TS rat model to evaluate the potential therapeutic effect of VOA in alleviating neuroinflammation in TS rats by modulating the p38 MAPK signaling pathway.

Tiapride, a selective D2 receptor (dopamine receptor) antagonist, has been used for years in the treatment of TS. Clinical and experimental studies have demonstrated that Tiapride has significant anti-tic effects (Chen, 2019; Pringsheim et al., 2019; Fekete et al., 2021). *Acorus tatarinowii* is a traditional medicinal botanical drug with a long history of use, known for its ability to restore vitality in the brain, nervous system, and cardiomyocytes, as well as its neuroprotective effects. It is widely used in the treatment of central nervous system, cardiovascular, and cerebrovascular diseases, and also exhibits antibacterial and antioxidant properties (Bai et al., 2022). The anti-inflammatory effects of essential oil metabolites in VOA have been demonstrated in both *in vitro* and *in vivo* studies against various stimulus-induced inflammatory responses (Zhong et al., 2020; Kim et al., 2022). Among these metabolites, 3-Methyl-pyrrolo [2,3-b]pyrazine exhibits various biological activities, including antibacterial, anti-inflammatory, antiviral, antifungal, antioxidant, antitumor, and kinase inhibition effects (Dehnavi, 2021). In a 90-day subchronic study with oral administration of α-Irone in 15 male and 15 female rats, no evidence of adverse toxic effects was observed upon necropsy (Oser et al., 1965). A dose of 0.4 g/kg of 3-Methyl-pyrrolo [2,3-b]pyrazine has been identified as safe in preliminary toxicity screenings (Stoner et al., 1973).1-Phenylcyclohexylamine shows potent anticonvulsant properties and is capable of inducing cumulative neuroplastic changes and alterations in brainwave patterns (Abiero et al., 2021; Thurkauf et al., 1990). β-asarone demonstrates multiple pharmacological activities, including antioxidant, anti-inflammatory, anti-apoptotic, anticancer, and neuroprotective effects (Meng et al., 2021; Balakrishnan et al., 2022). A subacute *in vivo* toxicity study indicated that oral administration of β-asarone (100 mg/kg for five consecutive days) led to reduced body weight and food consumption without causing mortality in rats prior to withdrawal (Balakrishnan et al., 2022). Citral, a monoterpene, has been extensively studied for its neuroprotective effects (Shamsi et al., 2024). It also exhibits antipyretic, antitumor, antibacterial, and anti-inflammatory activities (Dudai et al., 2005; Chaouki et al., 2009; Bachiega and Sforcin, 2011; Yang et al., 2013; Emílio-Silva et al., 2017.), inhibiting oxidative activity and enhancing brain-derived neurotrophic factor expression while reducing COX-2 and NF-κB expression and suppressing microglial activation (Katsukawa et al., 2010; Yang et al., 2013; Gonçalves et al., 2020; Charret et al., 2021; Habib et al., 2021). A 14-day toxicity assessment in male mice confirmed Citral’s safety, supporting its potential as a promising, innovative, and safe molecule for treating immune-inflammatory conditions and pain states (Gonçalves et al., 2020). Geraniol, an acyclic monoterpene derived from plants, has demonstrated anti-inflammatory, anticancer, antioxidant, and antibacterial effects in various *in vivo* and *in vitro* models (Rajendran et al., 2025). Studies suggest that Geraniol holds promise as a potential anti-inflammatory and cancer chemopreventive agent (Ben Ammar, 2023), exerting anti-inflammatory and immunomodulatory effects by reducing the expression of IL-6, IL-8, IL-1β, COX-2, NF-κB, and TNF-α (Wang et al., 2016; Ye et al., 2019; Pandur et al., 2024). Additionally, it reduces the expression of MAPKs (such as p38 MAPK), inhibiting microglial cells and neuroinflammation (Pandur et al., 2024; Rajendran et al., 2025). Trans-Calamenene, a metabolite contributing to the aroma and bioactivity of essential oils, exhibits antibacterial properties and may be considered as a candidate for natural antimicrobial agents (Gooré, 2017). Cis-Calamenene, the cis isomer of Calamenene, influences the maturation and function of human monocyte-derived dendritic cells, playing a role in immunomodulation by driving Th1 polarization, which is crucial for cellular immune responses, particularly in anti-infection and antitumor immunity (Takei et al., 2006).

Nevertheless, the potential toxicity of these metabolites must be acknowledged. The primary toxic metabolites of *A. tatarinowii* are the asarones found in its volatile oil, which have been shown to exhibit cardiotoxic, hepatotoxic, reproductive toxic, and carcinogenic effects (Jaiswal et al., 2015; Cartus and Schrenk, 2016; Bai et al., 2022). Specifically, studies indicate that doses of β-asarone above 500 mg/kg can result in acute mortality in mice, with toxicity increasing in a dose-dependent manner. Therefore, the use of essential oil must be strictly controlled within a safe dosage range. Precise regulation of the dosage, administration time, syndrome differentiation, and target system of the essential oil is crucial to prevent potential harm to humans. In this study, the therapeutic dose was based on clinical standards, calculated according to a 25 kg child’s body weight, with a clinical dose of 8g of *A. tatarinowii,* and was then converted to a corresponding dose for rats to ensure the safety and efficacy of the experimental research. Therefore, in this study, we used Tiapride as a positive control drug to compare its efficacy with that of VOA. In the motor and stereotyped behavior experiments, we found that both Tiapride and VOA significantly reduced the behavioral scores in TS rats.

Neuroinflammation refers to the inflammatory response within the central nervous system (CNS), characterized by elevated levels of pro-inflammatory cytokines, microglial activation, infiltration of peripheral leukocytes, and neuronal tissue damage (Estes and McAllister, 2014). In this process, microglia, the primary immune cells within the CNS, become activated in response to pathological stimuli and release large amounts of pro-inflammatory cytokines, such as TNF-α, IL-1β, and IL-6. These cytokines not only further activate microglia but also attract more peripheral immune cells to infiltrate the CNS, exacerbating neuroinflammation (Chen et al., 2019). For instance, in Parkinson’s disease (PD), neuroinflammation is considered one of the key factors driving neurodegeneration. Persistent microglial activation and excessive release of pro-inflammatory cytokines lead to the progressive loss of dopaminergic neurons, ultimately resulting in the onset and progression of motor symptoms. Microglia are key regulators of neuroinflammation, playing a significant role in the onset and progression of neurological diseases. Overactivated microglia are considered markers and drivers of neuroinflammation-related diseases (Whitney et al., 2009; Balakrishnan et al., 2022). *Postmortem* analyses of TS patients have shown increased numbers of CD45^+^ microglia in the striatum of the basal ganglia, with these cells exhibiting morphologies consistent with neurotoxic activation (Lennington et al., 2016). A recent PET study also demonstrated increased activation of microglia in the brains of TS patients (Kumar et al., 2015). In this study, we established a TS rat model using the neurotoxin IDPN. Through this model, we observed significantly elevated levels of pro-inflammatory cytokines, including TNF-α and IL-6, in the striatum of Model group rats, indicating a pronounced inflammatory response. Additionally, we detected significantly increased expression levels of microglial activation markers CD11 b and COX-2, further confirming the presence of an active microglial state and enhanced neuroinflammatory response in the TS rat model.

Fully activated microglia exhibit neurotoxic characteristics, specifically the M1 phenotype, producing pro-inflammatory mediators such as TNF-α, IL-1β, and IL-6. TNF-α and IL-6 are major stimulators of the inflammatory response in the CNS (Erta et al., 2012; Almolda et al., 2014; Recasens et al., 2021), possessing immunomodulatory and inflammation-regulating properties that can increase blood-brain barrier (BBB) permeability and promote neuronal death (Fujihara et al., 2020; Li et al., 2022). As a prototype immunoregulatory cytokine, IL-10 can control and modulate the production of pro-inflammatory cytokines both *in vitro* and *in vivo* (Ouyang et al., 2011; Leech et al., 2017), playing a crucial role in the interaction between the immune system and the nervous system (Long et al., 2019; Liu et al., 2021). It has been shown to induce the M2 phenotype of microglia or promote the conversion from M1 to M2, thereby effectively suppressing the production of inflammatory cytokines (Zhou et al., 2019; Cunha et al., 2022) and facilitating tissue remodeling and repair (Hoogland et al., 2015; Moehle and West, 2015; Tang and Le, 2016). We treated the Model group rats with VOA, Tiapride, and SB203580, respectively, and found that the levels of pro-inflammatory cytokines in the striatum of TS rats were significantly reduced after treatment, accompanied by decreased expression of the microglial marker CD11b, a reduction in the M1 marker COX-2, and an increase in the M2 marker CD163, indicating a transition and repair of neuroinflammation. Among these, VOA showed the most pronounced therapeutic effect, suggesting that VOA can effectively repair neuroinflammation.

The decrease in CD11 b and COX-2 levels in the striatum of TS rats further demonstrated VOA’s anti-inflammatory effects. CD11b, a microglia-specific surface marker protein, is a marker of pro-inflammatory polarization and is associated with the activation of M1 (pro-inflammatory) microglia. Increased expression of CD11 b correlates positively with the degree of microglial activation in various central nervous system diseases (Roy et al., 2008; Zhou et al., 2019; Liu et al., 2022). COX-2, a typical marker of pro-inflammatory cytokines, is an enzyme involved in the synthesis of pro-inflammatory prostaglandins. In the early stages of neuroinflammation, activated microglia mediate neuroinflammation and activate pro-inflammatory signaling pathways through COX-2 release (Aïd and Bosetti, 2011; López and Ballaz, 2020). Conversely, VOA treatment led to elevated levels of CD163, a hemoglobin-haptoglobin (HbHp) complex receptor, and a microglia-specific anti-inflammatory marker with strong antioxidant and anti-inflammatory properties. CD163 is a HbHp complex receptor and a microglia-specific anti-inflammatory marker with strong antioxidant and anti-inflammatory properties, playing a role in resolving inflammation and promoting tissue repair (Van Gorp et al., 2010; Pey et al., 2014; Sternberg et al., 2019; Han et al., 2022; Sharma et al., 2023). Consistent with these findings, we observed that VOA significantly downregulated CD11 b and COX-2 levels and increased CD163 levels in TS rats, indicating a downregulation of M1 pro-inflammatory mediators and an upregulation of M2 anti-inflammatory mediators, facilitating a reparative effect in the brains of TS rats. Notably, the increase in IL-10 further promoted the elevation of CD163 levels, both contributing to inflammation repair, adjusting the activation state of microglia, and alleviating neuroinflammation in TS rats.

Inflammation is characterized by membrane perforation mediated by the gasdermin protein family and sustained release of pro-inflammatory cytokines (Liu X. et al., 2016; Xu et al., 2018; Oh et al., 2020; De Schutter et al., 2021). Studies have shown that TNF-α can activate the NLRP3 inflammasome and caspase-1 (Yao et al., 2022). Activation of NLRP3 and caspase-1 in neurons leads to neuroinflammation (Hua et al., 2022). The NLRP3 inflammasome is a cytoplasmic protein complex composed of NLRP3, ASC, and Caspase-1 (Zhang et al., 2021), and its activation can lead to the proteolytic activation of Caspase-1 and increased secretion of pro-inflammatory cytokines IL-1β and IL-18, serving as a central signaling pathway for neuroinflammation (Kanthasamy et al., 2019; Weis et al., 2021). The NLRP3 inflammasome is one of the most widely studied and well-characterized inflammasomes in microglia. (De Rivero Vaccari et al., 2014; Han et al., 2021). NLRP3 activation is a crucial marker of M1 polarization and a key factor in inhibiting neuroinflammation (Han et al., 2023; Du et al., 2024), playing a critical role in microglial overactivation (Zhou et al., 2016; Dempsey et al., 2017; Mao et al., 2017). Its activation is closely related to neuronal damage in various neurological diseases (Wang Z. et al., 2019; Wang et al., 2022 M.). In this study, VOA intervention significantly reduced the levels of inflammatory cytokines such as TNF-α and IL-6 in TS rats, modulating the activation state of microglia and effectively regulating the neuroinflammatory state. The detection results showed that the decrease in the expression levels of NLRP3, a key marker of M1 polarization, and its downstream factors caspase-1 and GSDMD/GSDMD-NT proteins, also indicated that VOA could effectively reduce neuroinflammation in TS rats, serving as an effective regulation of M1 polarization.

p38 MAPK, the member of the MAPK family most closely associated with inflammation, is considered a central mediator of cellular signal transduction pathways and a key mediator of pro-inflammatory cytokine production (Cuenda and Rousseau, 2007; Bachstetter and Eldik, 2010; Meng et al., 2014; Li et al., 2018; Wang M. et al., 2022). It can mediate inflammatory responses and promote the activation of the NLRP3 inflammasome by inducing the production of inflammatory cytokines and chemokines (O’Neil et al., 2018; Wang S. et al., 2019; Hu et al., 2020), playing a crucial role in neurological diseases and CNS inflammatory responses (Liu et al., 2020; Liu et al., 2023). p38 MAPK is an important metabolite of autoimmune neuroinflammation (Motawi et al., 2023), acting as a significant mediator in signal transduction pathways involved in inflammation, cell cycle, cell death, cell differentiation, development, and tumorigenesis. Studies have shown that blocking the p38 MAPK signaling pathway attenuates the expression of pro-inflammatory cytokines and the NLRP3 inflammasome in inflamed mouse lung tissues. Pretreatment with SB203580 significantly reduced the expression of pro-inflammatory cytokines, IL-1β, TNF-α, and IL-6 by blocking the p38 MAPK signaling pathway (Li et al., 2018). p38 MAPK has anti-inflammatory functions through its negative regulation of the NLRP3 inflammasome, with a deficiency in p38 MAPK leading to upregulation of NLRP3 expression. Treatment with the p38 Inhibitor SB203580 can suppress the expression of NLRP3 inflammasome-related proteins (Liu et al., 2023). STAT3, a transcription factor, regulates cytokine-induced pro-inflammatory and anti-inflammatory responses, playing a key role in modulating microglia-induced inflammatory responses. Specifically, IL-6 can promote the induction of STAT3, leading to the expression of various genes involved in inflammation (Wu et al., 2019). Studies have shown that inflammatory factors like IL-1β and TNF-α can promote IL-6 secretion by upregulating NF-κB activity, thereby indirectly activating STAT3 (Liu F.-T. et al., 2016; Wu et al., 2019; Youssef et al., 2022). The p38 MAPK signaling pathway can regulate the downstream NF-κB pathway, mediating the gene expression of NLRP3 and related inflammatory factors (Huo et al., 2023). Treatment with the p38 MAPK Inhibitor SB203580 reduced levels of p-STAT3, TNF-α, and IL-10 after LPS stimulation. Inhibition of p38 MAPK can prevent STAT3 phosphorylation, indicating an interaction between the STAT3 and MAPK signaling pathways (Meng et al., 2014). As a key regulator of STAT3 phosphorylation, inhibiting p38 MAPK can reduce TNF-α secretion, weaken the inflammatory response, and completely block STAT3 phosphorylation, allowing the p38 MAPK-mediated STAT3 pathway to exert anti-inflammatory and antioxidant effects. Reduced STAT3 activity may also lead to decreased expression of downstream inflammatory mediators such as COX-2 and TNF-α, which were also found to be reduced in VOA-treated rats. Blocking the p38 MAPK signaling pathway resulted in decreased secretion of TNF-α by microglia, enhancing its protective effects. Therefore, inhibiting p38 MAPK phosphorylation can reduce NLRP3 protein expression, decrease TNF-α release, lower the expression of pro-inflammatory proteins COX-2 and CD11b, suppress IL-6 release, and increase levels of IL-10 and CD163, thereby inhibiting STAT3 activation and exerting inhibitory effects on microglia-induced neuroinflammation. Our findings are consistent with the above observations. Specifically, in the TS rat model, upregulation of the p38 MAPK signaling pathway was observed, followed by elevated levels of pro-inflammatory cytokines (IL-6 and TNF-α), activation of NLRP3, leading to the proteolytic activation of Caspase-1 protein, accompanied by increased expression of STAT3 protein. Correspondingly, under VOA treatment, levels of inflammatory cytokines (IL-6 and TNF-α) were reduced, expression of pro-inflammatory proteins COX-2 and CD11 b decreased, and levels of IL-10 and CD163 increased. This was accompanied by the attenuation of neuroinflammation and decreased expression of involved inflammatory signaling pathways p38 MAPK/NLRP3/STAT3.

## 5 Conclusion

**FIGURE 12:**
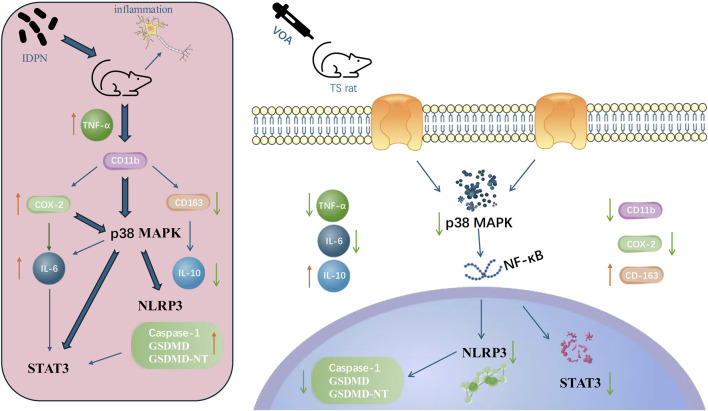
The possible mechanism of VOA in the treatment of TS.

In summary, our research indicates that VOA reduces the levels of pro-inflammatory cytokines and increases the concentration of anti-inflammatory cytokines in the striatum of TS rats, thereby influencing the levels of the NLRP3 inflammasome *in vivo*. Furthermore, our study reveals that VOA significantly diminishes microglial activation and the number of pro-inflammatory cytokines in the striatum of TS model rats. It also inhibits the activity of the p38 MAPK signaling pathway, the activation of the NLRP3 inflammasome, and the phosphorylation of STAT3, suggesting a neuroprotective role for VOA in the striatum of TS model rats. The potential mechanism of VOA in the treatment of TS is shown in Figure 12.

Notably, the innovation of this study lies in investigating the effects and potential mechanisms of VOA on neuroinflammation in the striatum of TS rats. Firstly, we found that VOA downregulates neuroinflammatory responses induced by the neurotoxin IDPN by inhibiting the p38 MAPK signaling pathway and the NLRP3 inflammasome associated with STAT3. Additionally, we demonstrated that VOA regulates neuroinflammatory responses by affecting STAT3 phosphorylation. Secondly, for the first time, we showed that VOA modulates the levels of inflammatory cytokines COX-2, CD11b, CD163, TNF-α, IL-6, and IL-10 in TS rats. Interestingly, we found that the combined application of VOA and the Inhibitor SB203580 more effectively inhibits the activity of the p38 MAPK signaling pathway compared to VOA alone, suggesting that VOA may exert its neuroprotective effects by inhibiting the p38 MAPK signaling pathway. Lastly, our study is the first to evaluate the impact of VOA on p38 MAPK/NLRP3/STAT3 signaling pathway-mediated neuroinflammation in the striatum of TS model rats.

Overall, our findings suggest that VOA exhibits a novel inhibitory effect on neuroinflammation mediated by the p38 MAPK/NLRP3/STAT3 signaling pathway in the TS model. This discovery lays the groundwork for considering VOA as a potential therapeutic candidate for treating neuroinflammation-related diseases, such as TS. However, this study did not explore the combined effects of Tiapride and VOA. Additionally, although the clinical safety range of VOA has been established, further studies on its toxicological profile are warranted. These areas of research may lead to new breakthroughs in the treatment of TS and will be further investigated in future studies.

## Data Availability

The original contributions presented in the study are included in the article/supplementary material, further inquiries can be directed to the corresponding authors.

## Glossary

AD: Alzheimer’s disease
ASC: Apoptosis-associated speck-like protein containing a CARD
ASD: Autism spectrum disorder
ANOVA: Analysis of Variance
BBB: Blood-brain barrier
caspase-1: Cysteine-aspartic acid protease-1
CD11b: Cluster of differentiation 11 b
CD163: Cluster of differentiation 163
**CD45** ^**+**^: Cluster of differentiation 45
CNS: Central nervous system
COX-2: cyclooxygenase-2
CTD: Chronic Tic Disorder
DNA: Deoxyribonucleic acid
DAPI: 4′,6-diamidino-2-phenylindole
DAB: Diaminobenzidine
ELISA: Enzyme-linked immunosorbent assay
ECL: Enhanced Chemiluminescence
GSDMD: Gasdermin D
GSDMD-NT: Gasdermin-D N-terminal fragment
GC-MS: Gas chromatography-mass spectrometry
HbHp: Hemoglobin-Haptoglobin
IDPN: 3,3′-iminodipropionitrile
IL-10: Interleukin-10
IL-18: Interleukin-18
IL-1β: Interleukin-1β
IL-6: Interleukin-6
LSD: Least Significant Difference
M1: Classically Activated Microglia
M2: Alternatively Activated Microglia
MAPK: Mitogen-Activated Protein Kinase
NF-κB: Nuclear factor kappa-B
NLR: Nod-like receptor
NLRP3: NOD-like receptor family, pyrin domain-containing 3
p38 MAPK: p38 Mitogen-Activated Protein Kinase
p-p38 MAPK: Phosphorylated p38 Mitogen-Activated Protein Kinase
p-STAT3: Phosphorylated Signal Transducer and Activator of Transcription 3
PD: Parkinson’s disease
PTD: Paroxysmal Tonic Downturn
PBS: Phosphate Buffered Saline
PVDF: Polyvinylidene fluoride Membrane
PET: Positron Emission Tomography
RNA: Ribonucleic acid
Rabbit SP: Rabbit Streptavidin-Peroxidase
RT-qPCR: Quantitative Real-Time Polymerase Chain Reaction
SD: Sprague-Dawley
STAT3: Signal Transducer and Activator of Transcription 3
Streptavidin-HRP: Streptavidin-Horseradish Peroxidase
TD: Tic Disorder
TNF-α: Tumor necrosis factor alpha
TS: Tourette syndrome
TBST: Tris Buffered Saline with Tween 20
VOA: The Volatile Oil of *Acorus tatarinowii*
